# In Vitro Wear Simulation of a Kinematically Aligned Knee Implant Tilted at an Angle of 8°

**DOI:** 10.1002/jor.70265

**Published:** 2026-08-17

**Authors:** Stefan Schroeder, Mareike Mueller, Maximilian Uhler, Therese Bormann, Sebastian Jaeger, Jan Philippe Kretzer

**Affiliations:** ^1^ Research Center of Biomechanics and Implant Technology, Department of Orthopaedics Heidelberg University Hospital Heidelberg Germany

**Keywords:** crosslinked polyethylene, kinematic alignment, knee wear simulation, mechanical alignment

## Abstract

Despite promising results of kinematic alignment (KA), there are still concerns regarding the wear behavior of KA knee implants. In a previous study, mechanically aligned (MA), KA and malaligned (MalA) knee implants were tested in a wear simulation study using Attune knee implant systems. The results showed no increased wear rate for a mild KA (KA4°) and MalA (MalA4°) condition in comparison to MA (MA0°) knee implants. This study analyzes the wear behavior of a KA condition of 8° (KA8°). The test setup was adapted accordingly. In addition, all tests of the previous study (MA0°, MalA4° and KA4°) and the KA8° condition were replicated using PFC implant systems to indicate if the previous results can be transferred to the predecessor model of the Attune knee implant system. The KA8° using the Attune knee implant system did not lead to a higher wear rate (2.4 ± 0.1 mg/10^6^ cycles) in comparison to the wear results of the previous study (3.8 ± 0.5 mg/10^6^ cycles for MA0°; 2.7 ± 0.2 mg/10^6^ cycles for MalA4° and 4.1 ± 0.2 mg/10^6^ cycles for KA4°). The PFC implant system led to higher wear rates than the Attune implant system but showed the same trend for the different alignment methods (5.1 ± 0.4 mg/10^6^ cycles for MA0°; 3.9 ± 0.1 mg/10^6^ cycles for MalA4°, 5.1 ± 0.8 mg/10^6^ cycles for KA4° and 3.5 ± 1.0 mg/10^6^ cycles for KA8°). KA8° did not show wear related problems but further in vitro and in vivo studies are necessary.

## 1 Introduction

Mechanical alignment (MA) is still the gold standard for the implantation of artificial knee joints [1]. Unfortunately, up to 20% of patients are unsatisfied with their replaced joint [2]. One hypothesis is that the MA operation method does not restore the natural joint line of the patient and thus, adjustments of the soft tissue are often necessary [3]. Soft tissue imbalance is associated with a high patient dissatisfaction [4]. To address this issue, an alternative approach evolved, the kinematic alignment (KA), with the goal of restoring the joint line [5]. For this purpose, patient‐specific bone cuts are carried out by the surgeon and implant components were placed according to the prearthritic state of the patient [6]. This surgical technique leads to similar results as MA regarding the revision rates [7, 8]. However, in the current literature, most studies either show an advantage of the KA technique or no difference in functional outcomes and patient satisfaction [8, 9, 10]. Despite these positive short‐term to mid‐term results the KA technique is controversially discussed. Especially the fact that femoral and tibial components are often implanted with a high angular varus‐valgus tilt relative to the mechanical axis using the KA technique, could lead to a significantly higher wear rate of the polyethylene inserts than the MA technique. Previous studies already reported an increased failure rate of implants for varus tilted components [11, 12]. A higher wear rate is inevitably accompanied by a higher number of released polyethylene particles, which can lead to aseptic implant loosening in the long‐term [13, 14]. Thus, it is highly relevant to analyze the wear behavior of KA in comparison to MA technique in a standardized approach. In a previous study, we addressed this topic and developed a test setup to compare the in vitro wear behavior of mechanically aligned knee implants, kinematically aligned knee implants with a tilted joint line by 4° and malaligned knee implants by 4° [15]. For more detailed information on the test conditions, we refer to our previous study [15]. In this study no difference could be found between the wear rate of KA and MA. However, a joint line tilt of 4° is only slightly outside the safe zone for surgeons using the MA technique. Therefore, the goal of this study is to address the following three research questions:

Does a KA condition with a tilted joint line of 8° lead to an increased wear behavior compared to the conditions from the previous study?

Are there different relative results between the alignment techniques, when using another knee implant system?

Do the implant designs differ regarding the wear rate, particle characteristics, joint kinematics and worn areas?

## 2 Methods

In a previous in vitro wear simulation study, a mechanical alignment condition of 0° (MA0°), a kinematic alignment condition of 4° (KA4°) and a malalignment condition of 4° (MalA4°) were compared regarding the wear rate, worn traces on the polyethylene inserts and joint kinematics (internal‐external rotation and anterior‐posterior translation) [15]. The MA0° condition was simulated by performing a standard wear simulation test. The joint line of the implant system is perpendicular to the compression force axis and coincides with the simulator axis. The KA4° condition was simulated by tilting the implant together with the simulator axis by 4°. The MalA4° condition was simulated by tilting the implant by 4° without tilting the simulator axis. Therefore, the implant axis and simulator axis of the malalignment condition do not coincide. The first part of the current study focuses on the wear behavior of a kinematic alignment condition of 8° (KA8°), which can be compared to the previous results. In the second part of the current study all tests of the previous study and the additional kinematic alignment test of 8° of the current study were repeated using the PFC Sigma implant system, which is the predecessor of the Attune implant system.

### 2.1 Tested Implants

To simulate the KA8° condition, three Attune® cruciate‐retaining implant systems (DePuy Synthes, Warsaw, Indiana, USA), with CoCr‐femoral components, fixed bearing CoCr‐trays and crosslinked polyethylene inserts blended with antioxidants and a thickness of 5 mm (size 5 of each implant component) were used as in the previous study. One additional implant system was used as loaded soak control.

For the repeating test of all conditions (MA0°, MalA4°, KA4°, KA8°) the PFC cruciate‐retaining implant system (DePuy Synthes, Warsaw, Indiana, USA), with CoCr‐femoral components, fixed bearing titanium trays and moderately crosslinked polyethylene inserts having a thickness of 10 mm (size 3 of each implant component) were used. For each of the four alignment conditions three PFC implant systems were tested and one additional implant system was used as loaded soak control. The size of the Attune implant system (size 5) is comparable to the size of the PFC implant system (size 3).

### 2.2 Test Setup

**Figure 1:**
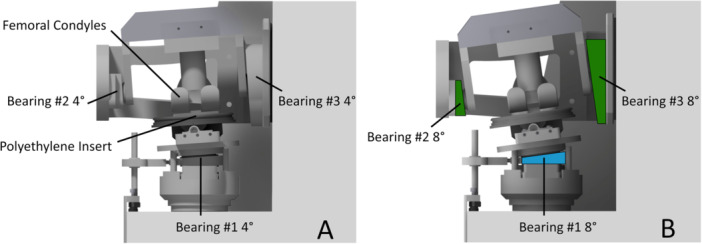
Illustration of the setup to simulate the kinematic alignment condition of 4° from the previous study (A) [15]. Test setup to simulate the kinematic alignment condition of 8° (B). The blue marked part shows the replaced hollow cylinder to incline the inner‐outer rotation by 8° and the green marked parts represent the replaced bearing bushings to incline the flexion‐extension motion by 8°.

The test setup to simulate the MA0° condition, the MalA4° condition and the KA4° condition is explained in detail in the previous study [15]. In order to simulate the KA8° condition, some parts of the test setup to simulate the KA4° condition had to be changed. The changed parts were the tilted bearings to apply the internal‐external rotation and the tilted bearings to apply the flexion‐extension motion as shown in Figure 1.

**Figure 2:**
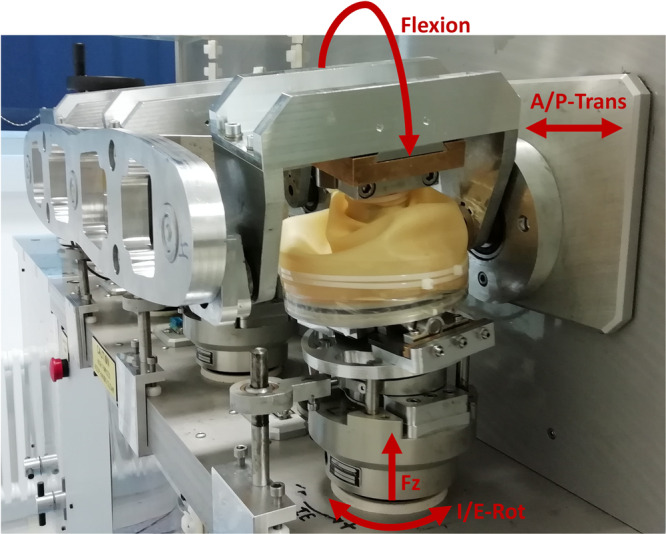
Wear simulator with the setups and implant systems to simulate the kinematic alignment condition of 8° (KA8°).

The tilted hollow cylinder with an angle of 4° was replaced by a tilted hollow cylinder with an angle of 8° (blue marked in Figure 1). The two angular bearing bushings of 4° to apply the flexion‐extension motion were replaced by angular bearing bushings of 8° (green marked in Figure 1). Three of the setups shown in Figure 1 were placed into the three test stations of an AMTI knee wear simulator (KS2‐6‐1000, Watertown, MA, USA) and an additional reference setup was placed in the reference station of the wear simulator. Figure 2 shows the knee wear simulator including the three test stations and the reference station.

### 2.3 Test Conditions

Before the start of the wear simulation, the polyethylene inserts were artificially aged for 2 weeks according to ASTM F2003‐2 [16]. Subsequently, the inserts were soaked in the fluid test medium for a minimum time of 10 weeks until the inserts were saturated [17]. Saturation was reached, when the weight changes within 24 h of the polyethylene inlays was below 10% compared to the previously measured weight changes. As in previous studies, force‐controlled wear simulations according to ISO 14243‐1 were performed to analyze the wear behavior of the KA8° condition using the Attune knee implant systems and to analyze the wear behavior of MA0°, MalA4° KA4° and KA8° conditions using the PFC knee implant system [18].

Therefore, the implant components were fixated into the test setups using polyurethane and test chambers were sealed and filled with 250 mL of fluid test medium each. The fluid test medium was bovine calf serum diluted to a protein content of 20 g/l and contains 1.85 g/l sodium azide and 5.85 g/l ethylenediaminetetraacetate to prevent bacterial growth and minimize calcium phosphate films on the surface of the implants. For each of the five tests 3.0 × 10^6^ cycles were performed and each 0.5 × 10^6^ cycles the test chambers were disassembled, cleaned and wear measurements were performed.

### 2.4 Wear and Kinematic Measurements

Wear and kinematic measurements were performed using different approaches. To analyze the wear performance, gravimetric measurements and particle analyses were used. To determine the joint kinematics, the AP‐translation and IE‐rotation were measured. Measurements of the worn areas on the polyethylene inserts were performed to get additional information about the wear behavior and the contact between femoral condyles and polyethylene insert during motion. Therefore, the worn areas were marked after wear simulation, photographed and the sizes of the marked areas were determined using ImageJ (version 1.49, National Institutes of Health).

### 2.5 Gravimetric Wear Measurements

To determine the polymeric wear rate, the polyethylene inserts were cleaned according to ISO 14243‐2 every 0.5 × 10^6^ cycles [19]. Afterwards a high precision balance (Sartorius Genius ME235S‐OCE, Sartorius AG, Goettingen, Germany) was used to measure the weight change of the three test inserts and the reference insert relative to the weights of the previous measurement interval. The weight gain of the reference insert was added to the absolute wear rate to account for soaking effects during wear simulation.

### 2.6 Particle Analysis

The test fluid after the test interval between 1.5 − 2.0 × 10^6^ cycles was used to characterize the released polyethylene particles according to the parameters described in ASTM F1877‐16 [20]. The first parameter was the Equivalent Circle Diameter (ECD), describing the diameter of a circle with the same area as the particle area and is therefore a measure of size. The Aspect Ratio (AR) describes the maximum distance between any two points on the particle outline (D_max_) divided by the maximum distance between any two points on the particles outline perpendicular to D_max_. The AR was also used to determine the amount of round (AR < 1.5) oval (1.5 ≤ AR ≤ 2.5) and fibril‐like particles (AR > 2.5) similar as Catelas et al. did for metal wear [21]. The roundness (R) has values between zero and one, whereby a low value indicates an elongated particle and a value of one corresponds to a completely round particle. To determine these parameters, the test fluids of the three test chambers were mixed and a digestion process was performed as described in previous studies [22, 23]. Afterwards, the resulting fluid was filtered through alumina filters with a pore‐size of 0.1 µm (AnodiscTM 13, WhatmanTM, GE Healthcare Life Sciences, Amersham, UK). Filters with a pore‐size of 0.1 µm were used because the majority of the expected polyethylene particles is typically bigger than the used pore‐size and the most biological active polyethylene particles are between 0.1 and 10 µm in size [14, 24]. Then, electron microscopy (FEGSEM, LEO 1530, Leo, Oberkochen, Germany) with a magnification of 10.000× was used in order to take images, which could be analyzed using a digital image software (Particleanalyzed_HD, LBI, Heidelberg, Germany) [25]. For each wear simulation group, three filtrations were carried out and three images were made from each filter. Thus, in total nine images were made for each group. Particle analyses were also performed using the frozen test fluid from the previous study analyzing the MA0°, MalA4° and KA4° conditions of the Attune implant groups.

### 2.7 Joint Translation and Rotation

Due to the force‐controlled wear simulation, a force was applied in the anterior‐posterior direction and an internal‐external torque was applied around the implant axis for the MA0°, KA4° and KA8° condition. For the MalA4° condition, the internal‐external torque was applied around the simulator axis, but not around the implant axis, due to the tilt of 4°. The cardanics in the KA conditions led to a redirection of the torque around the simulator and the implant axis. Therefore, the AP‐motion and IE‐rotation result from the implant design and/or implant orientation. These two motions were recorded by the software of the simulator and analyzed after 0.25 × 10^6^ cycles of each interval for every test group.

### 2.8 Worn Areas

The worn areas of the polyethylene inserts were determined by outlining these areas and subsequent imaging of the inserts. The areas of the worn surface were then measured using ImageJ (Version 1.49, National Institutes of Health, Bethesda, MD, USA).

### 2.9 Statistics

For the comparison of the wear rates, regression analyses were used between 0.5 and 3.0 × 10^6^ cycles. The parameters for particle analysis (ECD, AR and R), the joint kinematics and the wear traces were descriptively evaluated by arithmetic mean and standard deviation. Afterwards the results (wear rate, particle characteristics, joint kinematics and total worn areas) of the five groups of the current study (KA8° of the Attune knee implant system and MA0°, MalA4°, KA4° and KA8° of the PFC implant system) and the three groups of the previous study (MA0°, MalA4° and KA4° of the Attune knee implant system) [15] were compared to each other using two‐way ANOVA for independent samples and, if applicable, Bonferroni post‐hoc analysis. A *p*‐value of ≤ 0.05 was considered significant. All statistical analyses were carried out using SPSS 22 (IBM, Amonk, NY, USA).

## 3 Results

### 3.1 Gravimetric Wear Rates

**Figure 3:**
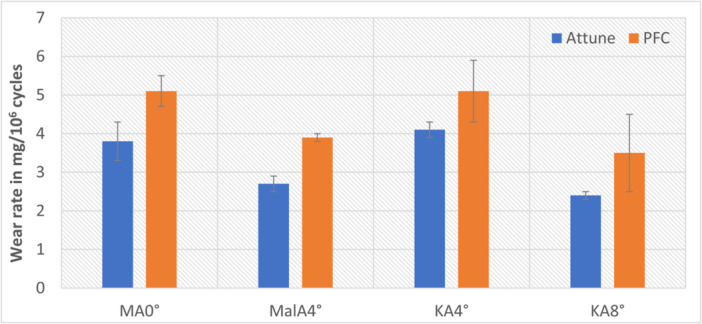
Wear rates of the four alignments of both groups.

The wear rates per 10^6^ cycles are shown in Figure 3.

The two‐way ANOVA showed a significant difference between the four alignments (*p *< 0.001). The results of the post‐hoc analyses are summarized in Table 1.

**Table 1:** *p*‐values and 95% Confidence Interval between the different alignments using the two‐way ANOVA.

Alignment	MA0°	MalA4°	KA4°	KA8°
MA0°	—	—	—	—
MalA4°	*p* = 0.009 [0.51, 1.79]	—	—	—
KA4°	*p* > 0.99 [−0.76, 0.52]	*p* = 0.004 [−1.91, −0.63]	—	—
KA8°	*p* = 0.001 [0.82, 2.11]	*p* > 0.99 [−0.32, 0.96]	*p* < 0.001 [0.94, 2.22]	—

In addition, a significantly lower wear rate was found for the Attune knee implant system compared to the PFC implant system for all alignments (95% CI [0.70, 1.60], *p* < 0.001). There was no significant interaction between the factors alignment and implant system for the wear rate (*p* = 0.964).

### 3.2 Particle Characteristics

**Figure 4:**
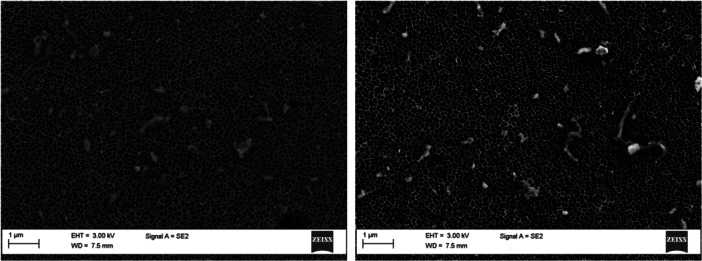
Exemplary electron microscopy images of the Attune (left) and PFC (right) implant system.

The particle characteristics of the four alignments of both implant systems are summarized in Table 2. In Figure 4, two exemplary electron microscopy images of the Attune and PFC implant systems are shown.

**Table 2:** Particle characteristics (ECD, AR and R) with 95% Confidence Interval of the four alignments of both implant systems.

Implant	Factor	MA0°	MalA4°	KA4°	KA8°
Attune	ECD in µm 95% CI	0.21 ± 0.05 [0.19, 0.23]	0.18 ± 0.02 [0.16, 0.20]	0.19 ± 0.02 [0.17, 0.21]	0.22 ± 0.03 [0.20, 0.23]
	AR 95% CI	2.04 ± 0.21 [1.87, 2.22]	2.06 ± 0.21 [1.88, 2.23]	2.04 ± 0.09 [1.86, 2.21]	2.17 ± 0.33 [1.99, 2.34]
	R 95% CI	0.59 ± 0.02 [0.57, 0.62]	0.58 ± 0.02 [0.55, 0.60]	0.58 ± 0.01 [0.56, 0.61]	0.57 ± 0.02 [0.55, 0.60]
PFC	ECD in µm 95% CI	0.24 ± 0.03 [0.22, 0.26]	0.26 ± 0.02 [0.22, 0.26]	0.26 ± 0.05 [0.24, 0.28]	0.24 ± 0.02 [0.24, 0.28]
	AR 95% CI	2.07 ± 0.11 [1.90, 2.25]	2.12 ± 0.26 [1.95, 2.30]	2.08 ± 0.44 [1.90, 2.25]	2.23 ± 0.25 [2.05, 2.40]
	R 95% CI	0.56 ± 0.03 [0.54, 0.59]	0.56 ± 0.04 [0.53, 0.58]	0.56 ± 0.07 [0.53, 0.58]	0.55 ± 0.05 [0.53, 0.58]

**Figure 5:**
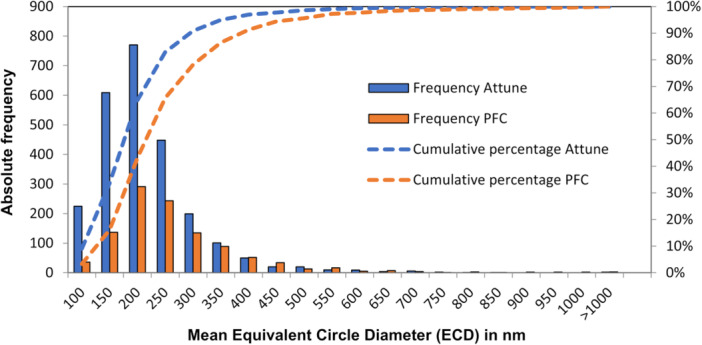
Frequency distribution of the Equivalent Circle Diameter (ECD) of the Attune and the PFC implant system.

Thereby, the mean number of particles per image analyzed for the Attune knee implant was 78 ± 34 for the MA0°, 63 ± 8 for the MalA4°, 86 ± 22 for the KA4° and 44 ± 17 for the KA8° group. The mean number of particles per image analyzed for the PFC knee implant was 40 ± 5 for the MA0°, 23 ± 5 for the MalA4°, 32 ± 9 for the KA4° and 24 ± 5 for the KA8° group. The numbers of particles analyzed per image for the PFC knee implant was lower compared to the Attune knee implant system due to a reduction of the concentration of the analyzed fluid sample. Regarding the ECD, the two‐way ANOVA did not show significant differences between the four alignments (*p *= 0.078). However, the PFC implant system led to significant larger particles compared to the Attune implant system (95% CI [38.98, 65.58], *p* < 0.001) as shown in Figure 5.

**Figure 6:**
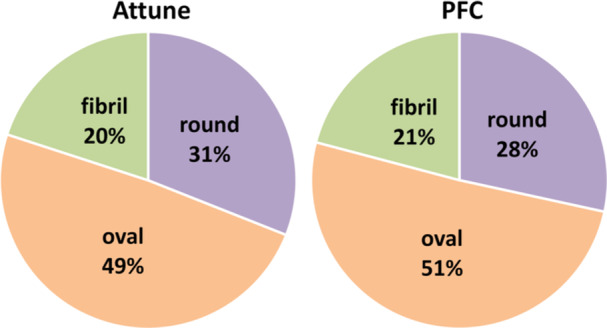
Classification of the resulting polyethylene particles into round, oval and fibril‐like of both implant systems.

Regarding the AR, the two‐way ANOVA did neither show significant differences between the four alignments (*p *= 0.336) nor between the two implant systems (95% CI [−0.08, 0.17], *p*= 0.434). In Figure 6, the percentages of round, oval and fibril‐like particles of each implant system are demonstrated.

Regarding the Roundness, the two‐way ANOVA did not show significant differences between the four alignments (*p* = 0.654). However, the Attune implant system led to significant rounder particles compared to the PFC implant system (95% CI [−0.04, −0.01], *p* = 0.007). No significant interactions were found between the factors alignment and implant system for the ECD (*p* = 0.209), AR (*p* = 0.997) and R (*p* = 0.996).

### 3.3 Comparison of the Joint Kinematics

**Figure 7:**
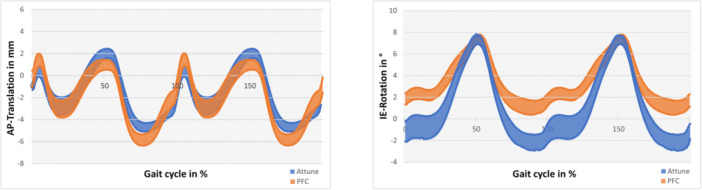
Kinematics (AP‐translation and IE‐rotation) over 200% of the gait cycles for both implant systems. Each curve represents the span of the four alignments.

**Figure 8:**
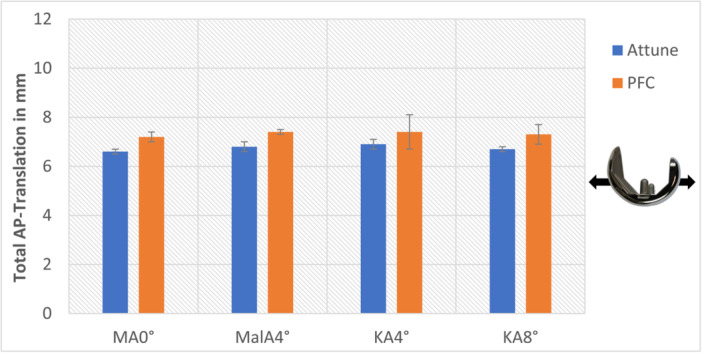
Total AP‐translations of the four alignments of both implant systems.

To compare the resulting joint kinematics of the alignment and implant groups the total AP‐translation and the total IE‐rotation between femoral condyle and tibia plateau were analyzed separately. The AP‐translation and IE‐rotation of the Attune and the PFC implant system over two gait cycles are illustrated in Figure 7. Thereby, each curve contains the span of the four alignments. The resulting total AP‐translations of the alignment and implant groups are shown in Figure 8.

No significant differences were found between the AP‐translations of the four alignments (*p* = 0.398). Comparing the two implant systems, the PFC implant system led to a significantly higher AP‐translation (95% CI [0.34, 0.69], *p* < 0.001). No significant interactions were found between the factors alignment and implant system for the AP‐translation (*p* = 0.062).

**Figure 9:**
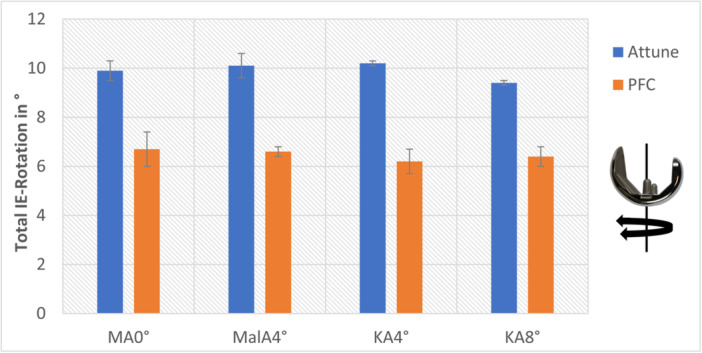
Total IE‐rotations of the four alignments of both implant systems.

The resulting total IE‐rotations (measured relative to the simulator axis) of the alignment and implant groups are shown in Figure 9.

No significant differences were found between the IE‐rotations of the four alignments (*p* = 0.066). Comparing the two implant systems, the Attune implant system led to significantly higher IE‐rotations (95% CI [−3.63, −3.14], *p* < 0.001). A significant interaction was found between the factors alignment and implant system for the IE‐rotation (*p* = 0.016).

### 3.4 Comparison of the Wear Traces

**Figure 10:**
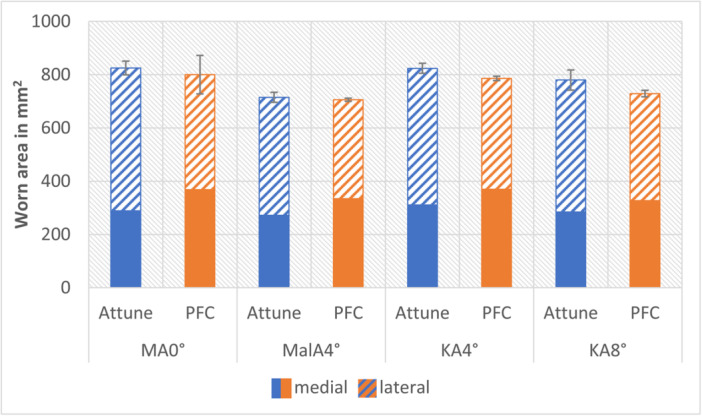
Worn areas of the medial and lateral side of the four alignments of both implant systems.

The worn areas on the medial and lateral side of the polyethylene inserts after the tests were shown in Figure 10.

**Figure 11:**
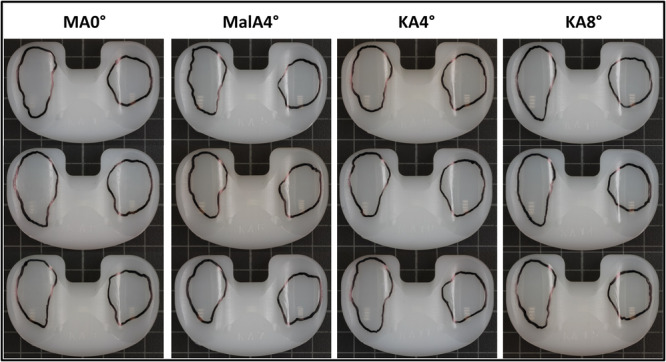
Worn areas of the four alignments (PFC implant system).

Comparing the total worn areas (medial + lateral side) between the four alignments, the MA0° condition led to larger worn areas than the MalA4° condition (95% CI [45.70, 157.30], *p *< 0.001) and the KA8° condition (95% CI [2.03, 113.64], *p* = 0.040). The KA4° condition led to larger worn areas compared to the MalA4° condition (95% CI [38.20, 149.80], *p* < 0.001), but no significant difference was found between the KA4° condition and the KA8° condition (95% CI [−5.47, 106.14], *p* = 0.092). In addition, no significant differences were found between the MA0° condition and the KA4° condition (95% CI [−48.30, 63.30], *p* > 0.99) as well as between the MalA4° condition and the KA8° condition (95% CI [−99.47, 12.14], *p* = 0.190). To illustrate the impact of the different alignments Figure 11 shows the worn areas of all test inserts of the PFC implant system. Comparing the two implant designs, the Attune knee implant system led to significantly larger worn areas than the PFC implant design (95% CI [3.11, 58.72], *p* = 0.031). No significant interactions were found between the factors alignment and implant system for the total worn areas on the polyethylene inserts (*p* = 0.713).

## 4 Discussion

In the current study the KA8° condition was added to the previous study results analyzing the wear behavior of MA0°, MalA4° and KA4° using the Attune knee implant. In addition, all wear simulations (MA0°, MalA4°, KA4° and KA8°) were repeated with another knee implant system (PFC) using the same approach. Thereby, the research questions could be answered as follows:

*Does a KA condition with a tilted joint line of 8° lead to an increased wear behavior compared to the conditions from the previous study?*

The KA8° condition did not lead to an increased wear behavior. Indeed, the wear rate of this alignment condition was lower than the MA0° condition or the KA4° condition and similar to the MalA4° condition. The particle characteristics and the joint kinematics were not significantly different between the KA8° and the other three alignment conditions. However, the worn areas on the polyethylene inserts were also smaller in the KA8° condition compared to the MA0° condition (significantly) and the KA4° condition (tendency). The worn areas of the KA8° and the MalA4° condition were not significantly different to each other.

Maybe the KA8° condition changes the contact area between femoral condyle and polyethylene insert during motion leading to a lower wear rate. Similar results were found for the MalA4° condition in the previous study. In an in vitro study by Brockett et al. the wear performance of two different conformities of the PFC Sigma polyethylene inserts were analyzed [26]. Thereby, the wear rates of lipped and flat (machined) inserts were compared using a knee wear simulation test. Although, only the worn areas were used to determine conformity changes instead of contact area and contact pressure measurements, it was shown, that a reduced conformity and thus a smaller contact area between femoral condyle and polyethylene insert leads to significantly lower wear rates. The reduced wear rate and worn areas of the KA8° group and MalA4° group might look beneficial in the first place. However, a more even and fuller contact between the implant components as for the MA0° and KA4° condition might be safer in the long‐term. The smaller contact areas of the KA8° condition and MalA4° condition could provoke higher peak contact pressures on the insert during motion, leading to possible delamination of the polyethylene after several years in vivo. To support this statement, contact pressure measurements are necessary and should be performed in a future study.

*Are there different relative results between the alignment techniques, when using another knee implant system?*

Looking at the non‐significant interactions between implant type and alignment, it can be followed, that the PFC implant system does not lead to different relative wear rates, worn areas and particle characteristics between the four alignments compared to the Attune implant system. Focusing on the joint kinematics, no significant interaction was found between implant type and alignment for the AP‐translation, but for the IE‐rotation. The interaction for the IE‐rotation can be explained by the KA4° condition. The KA4° condition led to a higher IE‐rotation than the other three alignment conditions for the Attune implant system and led to smaller IE‐rotation than the other three alignment conditions for the PFC implant system. However, the maximum range of the IE‐rotation between the four alignments was 0.8° for the Attune implant system and 0.5° for the PFC implant system, which is relatively low.

*Do the implant designs differ regarding the wear rate, particle characteristics, joint kinematics and worn areas?*

The wear rates of the PFC implant system were higher compared to the more modern Attune knee implant system although the worn areas on the inlays were smaller for the PFC. Regarding the joint kinematics, the PFC showed more translation in anterior‐posterior direction than the Attune implant system, but led to a smaller internal‐external rotation. The higher total internal‐external rotation of the Attune implant system of approximately 3° compared to the PFC implant system had a higher impact on the size of the worn areas compared to the relatively small difference regarding the anterior‐posterior translation. The difference in the joint kinematics between the two implant designs can be attributed to the fact, that the shapes of the femoral condyles and the troughs in the polyethylene inserts differ and due to the application of a force‐controlled wear simulation. Despite the smaller worn areas, the PFC implant system led to higher wear rates and to bigger particles. This effect can be attributed to the higher crosslinking and the addition of Vitamin E to the polyethylene inserts of the Attune knee implant system. These factors lead to a reduced wear rate and make the inlay robust against aging processes [27]. Previous studies already demonstrated that a higher crosslinking of polyethylene inserts is associated with smaller and rounder particles during articulation [28, 29]. Nonetheless, due to the different designs and different materials of the two implant systems, the impact of each factor cannot be determined.

### 4.1 Clinical Relevance

The maximum wear rate occurred by simulating the MA0° and KA4° conditions using the PFC implant system. Both conditions led to a mean wear rate of 5.1 mg/10^6^ cycles (5.1 ± 0.4 mg/10^6^ cycles for the MA0° and 5.1 ± 0.8 mg/10^6^ cycles for the KA4° condition). This wear rate corresponds to a volumetric wear rate of approximately 13 (95% CI [11, 14]) mm^3^/year taking a polyethylene density of 0.95 mg/mm^3^ and a yearly activity of 2.4 (95% CI [2.1, 2.6]) million steps into account [30]. None of the polyethylene inserts showed delamination. This can be attributed to the crosslinked polyethylene for the PFC implant system and the crosslinked polyethylene inserts enriched with antioxidants for the Attune implant system. Popoola et al. tested aged polyethylene inserts made from conventional polyethylene and aged crosslinked polyethylene inserts in a wear simulation study and a delamination test [31]. All of the conventional polyethylene inserts showed delamination and surface cracks but none of the crosslinked polyethylene inserts did. It can be assumed, that a kinematic alignment condition of up to 8° or a malalignment condition of up to 4° might not lead to wear related problems as long as modern crosslinked polyethylene is used and no edge‐loading occurs. Nonetheless, this statement must be treated with caution, as the study by Popoola et al. employed a crosslinking protocol distinct from that of the commercially available inlays used in the current study. Furthermore, additional tests using aged conventional polyethylene would be necessary to compare the results of the current study with the results of Popoola et al. However, the smaller worn areas of the KA8° and MalA4° conditions could indicate higher peak pressures which may lead to higher wear rates due to delamination in the long‐term. In addition, other non‐wear related problems, which were not subjected to this study may reduce the longevity of the implant for KA8° and MalA4° conditions.

### 4.2 Limitations

For in vitro wear simulation studies, some limitations need to be mentioned. Despite the possibility to apply a force‐controlled simulation with virtual ligaments to the implants, a wear simulation study cannot replicate the in vivo situation entirely. Only one specific implant orientation for each group could be analyzed and calf serum is used instead of human synovia fluid. However, this approach allows a standardized test method with just varying alignment conditions. Thereby, patient specific factors, which may influence the results, could be eliminated. Another important point, is that the force vector moves more laterally by applying higher angles in the KA conditions. This is due to an offset between the axis for the varus‐valgus rotation and the contact points between femoral condyles and polyethylene insert. To reduce the impact of the lateralization of the force, the offset was reduced to a minimum during the construction process of the test setup. The internal‐external moment is not affected by the offset and the force in anterior‐posterior direction. Another limitation are the limited alignment conditions that could be analyzed. Due to the fact, that kinematic alignment is a patient‐specific method indefinitely combinations of the implant orientations would be possible to simulate. We only focused on the varus tilt of the joint line for the kinematic alignment conditions and its influence on the wear behavior. Based on these results, further investigations would be useful including different slopes and combinations of tibial varus/valgus and femoral varus/valgus. Finally, with regard to the wear rates of the two implant systems, it is not clear to what extent kinematics and the different types of polyethylene contribute to the results. However, this could only be clarified if either the designs were identical and two different polyethylene materials were used, or vice versa.

## 5 Conclusion

The results of this study in combination with the results of the previous study show, that neither a KA condition of 8° nor a MalA condition of 4° led to an increased wear behavior. However, the reduced worn areas of the polyethylene inserts for the KA8° and MalA4° condition might come along with a higher contact pressure under articulation. This might lead to a higher potential of delamination of the polyethylene inserts compared to a mild kinematic alignment condition of 4° or a mechanical alignment condition of 0°. To support this statement, contact area and pressure measurements are necessary and should be performed in a future study.

## Author Contributions

**S. Schroeder** – conceptualization, validation, formal analysis, investigation, writing (original draft), project administration; **M. Mueller** – investigation, formal analysis, writing (review and editing); **M. Uhler** – conceptualization, project administration, writing (review and editing); **T. Bormann** – validation, formal analysis, writing (review and editing); **S. Jaeger** – validation, writing (review and editing); **J. P. Kretzer** – methodology, resources, writing (review and editing), supervision.

## Data Availability

The data that support the findings of this study are available from the corresponding author upon reasonable request.
